# Functional analysis implicating the SNP rs61552325 in ERBB2 as an effector for androgen-insensitive prostate cancer cell invasion

**DOI:** 10.18632/oncotarget.16807

**Published:** 2017-04-04

**Authors:** Xianxiang Xin, Yinmin Gu, Yang Chen, Yuanjie Huang, Zengnan Mo, Yanling Hu

**Affiliations:** ^1^ Experimental Centre of Medical Sciences, Guangxi Medical University, Nanning, Guangxi, China; ^2^ Commission for Discipline Inspection, Yantai Affiliated Hospital of Binzhou Medical University, Yantai, China; ^3^ Center for Genomic and Personalized Medicine, Guangxi Medical University, Nanning, Guangxi, China; ^4^ Guangxi Colleges and Universities Key Laboratory of Biological Molecular Medicine Research, Guangxi Medical University, Nanning, Guangxi, China

**Keywords:** prostate cancer, single nucleotide polymorphism, metastasis, androgen-insensitive

## Abstract

**Background:**

As one of the most common cancers in men, the pathogenesis of prostate cancer has been widely researched. Aberrant activation of the erb-b2 receptor tyrosine kinase 2 (ERBB2) has been found to play a critical role in metastatic prostate cancer. In our previous study, we demonstrated that rs61552325 (Pro1140Ala) located in ERBB2 is strongly correlated to prostate cancer. Therefore, we initially studied the effect of rs61552325 on androgen-independent prostate cancer cell metastasis.

**Results:**

Bioinformatic results demonstrated that the mutant Pro1140Ala likely decrease the stability of the ERBB2 protein and its interactions. The mean migration rate after 6 h for PC3 minor variant cells which carried the G allele was 1.28-fold higher than major variant PC3 cells that carried the C allele (*P* = 0.016). The mean invasion rate of DU145 putative minor variant cells was 0.40 reducer than negative control cells (*P* = 5.9E-04).

**Methods:**

rs61552325 major variant (C allele) and minor variant (G allele) were produced by site directed mutagenesis and transfected into DU145 and PC3 cells. A wound healing assay was performed to compare migration abilities between alleles. After knocking down endogenous ERBB2 and then expressing the rs61552325 minor variant, invasion abilities were evaluated with a transwell assay using DU145 and PC3 cells.

**Conclusions:**

Our data showed that the rs61552325 major variant decreases PC3 cell migration and its minor variant depresses DU145 cell invasion, suggesting that rs61552325 is likely an important change during prostate cancer invasion.

## INTRODUCTION

A recent study provided an evidence-based analysis for the epidemiology of prostate cancer (PCa). The global incidence of PCa has increased in the last 20 years as the population ages [1]. In 2008, a report of global statistics revealed that 903,500 men were diagnosed with and 258,400 men die of PCa by the age of 75 [2]. PCa can transfer to the brain and bones, and metastasis of PCa leads to poor prognoses and increases difficulty of disease treatment [3]. In one-on-one interviews with 25 PCa patients (18 metastatic), the major focus was on bone pain, urinary dysfunction, bowel dysfunction, and some psychotic symptoms [4]. These symptoms are a heavy burden for the patients, their family and society as a whole. Resolution of this problem is challenging, and researchers have pursued many avenues to uncover the mysteries of metastatic PCa.

It is widely accepted that most oncogenes are involved with metastatic PCa, including the erb-b2 receptor tyrosine kinase 2 (ERBB2) [5]. The ERBB2 proto-oncogene, known as HER2, is located on chromosome 17q21. It encodes a member of the epidermal growth factor receptor (EGFR) family of tyrosine kinase receptors and involves in many cell activities such as cell differentiation, proliferation and invasion [6–9]. ERBB2 can initiate a cascade of signaling pathways and active the survival factor AKT by forming heterodimers with other members of the ERBB family [10, 11]. Patients with hormone refractory PCa have higher levels of ERBB2, suggesting that increased ERBB2 correlates with the presence of castrate, metastatic PCa [12, 13]. Elevated ERBB2 in androgen-dependent PCa cells was sufficient to confer androgen-independent growth and accelerate progression to androgen-independence [14]. These findings indicated that ERBB2 may contribute to the disease progression of PCa to metastasis and androgen independence. Interestingly, ERBB2 has been implicated in increased metastatic potentials, specifically in androgen-insensitive PCa cells [5].

Genetic polymorphisms are the most common form of genetic variation. While the exact contributions of genetic factors to this cancer are obscure, single nucleotide polymorphisms (SNPs) have been associated with various cancers through interactions with environmental factors [15]. For example, rs652438 located in matrix metallopeptidase 12 (MMP-12) leads to a missense change of asparagine to serine [16]. rs2276109, an A-to-G substitution at position -82 in the MMP-12 promoter, contributes to an increase in MMP-12 transcription [17]. These examples support the idea that mutations in functional loci can alter the amino acid sequence and subsequently protein structure and gene function. Additionally, rs10486567 located in intron 3 of the JAZF zinc finger 1 (JAZF1) gene on 7 chromosome can affect both the NK3 homeobox 1 (NKX3-1) and the FOXA-AR motif in different alleles, resulting in a 39% increase in basal activity. After androgen stimulated enhancer activity, a 28% fold-increase can be found [18]. rs10993994 has a major effect on the transcriptional activity of microseminoprotein beta (MSMB), a suppressor gene in PCa, which increases the risk of PCa [19]. Research on the function of genetic polymorphisms might uncover the mysteries behind genes that cause cancer.

Our previous study found that rs61552325 located in ERBB2 is strongly related with PCa (*P* = 0.001) [20]. The rs61552325 in The Single Nucleotide Polymorphism database (dbSNP) has been merged with rs1058808, and is located in the coding region of ERBB2. The polymorphism rs61552325 changes an amino acid from proline to alanine. Some studies have shown that the rs61552325 polymorphism is associated with glioblastoma multiforme, endometrial cancer and asthma [21–23]. However, almost nothing is known about the functional significance of the ERBB2 rs61552325 polymorphism in PCa. Thus, we hypothesized that rs61552325 could alter the metastatic potential of androgen-insensitive PCa. In this study, we tried to investigate the biological function of rs61552325 on androgen-insensitive PCa cells by conducting a wound healing test and a transwell invasion assay *in vitro*. Our findings support the notion that the rs61552325 polymorphism plays a central role in the mechanism of metastasis for androgen-insensitive PCa.

## RESULTS

### *In silico* prediction of protein stability

The stability and pathogenicity of ERBB2 were predicted using four tools I-Mutant Suite [24–25], iStable [26], PolyPhen-2 [27] and HOIPE [28], and revealed that the mutation Pro1140Ala was very damaging and decreased protein stability (Table 1). Structural stability analysis performed by the I-Mutant Suite proposed a ‘decrease’ in the stability of the mutant protein as that the free energy changing value was -1.37, which was less than 1.0 kcal/mol. The iStable analysis also showed a ‘decrease’ in the structural stability of the mutant protein. The stability score predicted in PolyPhen 2 was 0.948, which was classified as “most likely damaging”. An analysis using HOPE revealed that the wild-type and mutant amino acids differed in size, and the mutant residue was smaller which might lead to loss of interactions. Additionally, the results showed that prolines had a very rigid structure which sometimes forced the protein backbone into a specific conformation in view that this mutation changed a proline to another residue that disturbed the local structure. These bioinformatic results demonstrated that the mutant Pro1140Ala likely decreases the stability of the ERBB2 protein and its interactions.

**Table 1:** Damaging effects prediction of rs61552325 using in *silico* tools

Prediction tool	Analyzing Method	interpretation
I-Mutant Suite [24–25]	Support Vector Machines and evolutionary information	Decrease (-1.37)
iStable [26]	Grid computing architecture constructed by using sequence information and prediction results from different element predictors	Decrease
PolyPhen 2 [27]	Eight sequence-based and three structure-based predictive features which were selected automatically by an iterative greedy algorithm	Most likely damaging (0.948)
HOPE [28]	Collects information from a wide range of information sources including calculations on the 3D coordinates of the protein by using WHAT IF Web services, sequence annotations from the UniProt database, and predictions by DAS services.	The mutant residue is smaller, this might lead to loss of interactions.Your mutation changes a proline with such a function into another residue, thereby disturbing the local structure.

### The rs61552325 major variant decreases PC3 cell migration

**Figure 1:**
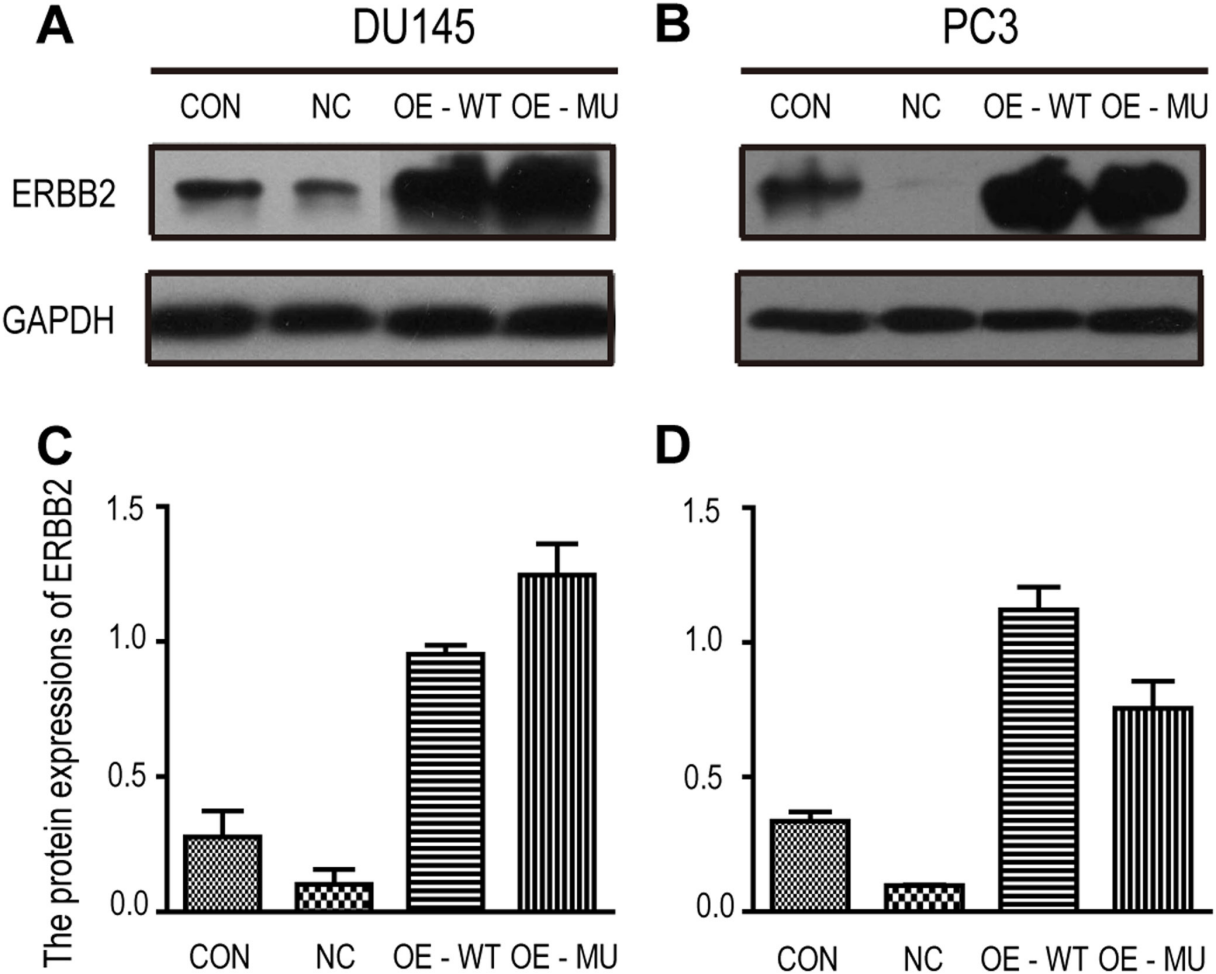
Western blot analysis of ERBB2 expression in DU145 and PC3 cells from the wound healing assay **(A)** Western blot of ERBB2 protein levels in DU145 cells. **(B)** Western blot of ERBB2 protein levels in PC3 cells. **(C)** Band intensities for ERBB2 were quantified and normalized to GAPDH. **(D)** ERBB2 protein expression in PC3 cells is presented as the ratio of the gray scales of ERBB2 bands and the gray scales of GADPH bands. Abbreviations: CON: control group, DU145 or PC3 cells without any viral infection; NC: negative control, DU145 or PC3 cells infected with the negative control virus; OE-WT: DU145 or PC3 cells infected with the major variant (C allele) virus of rs61552325; OE-MU: DU145 or PC3 cells infected with the minor variant (G allele) virus of rs61552325.

**Figure 2:**
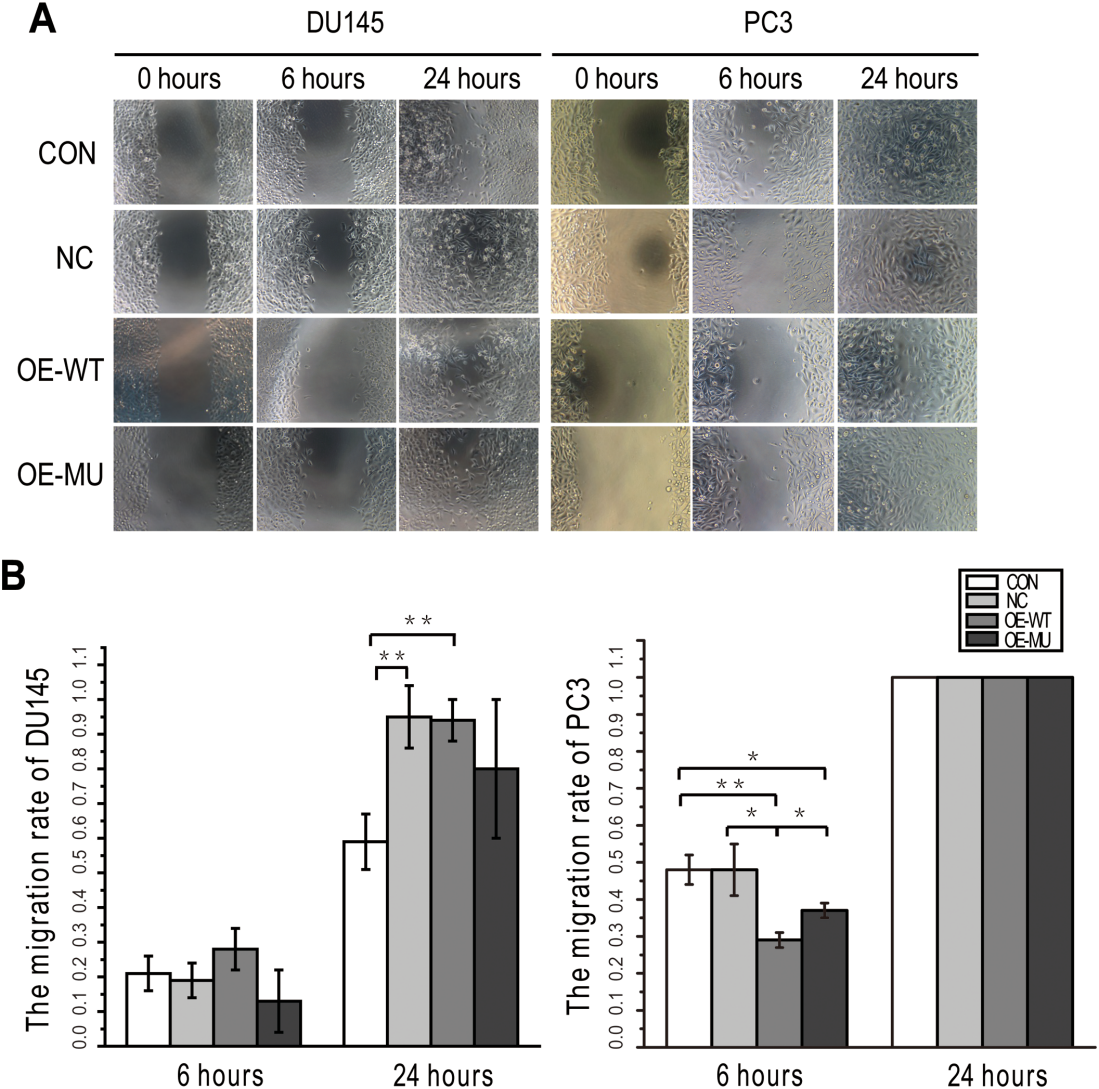
SNP rs61552325 effects on DU145 and PC3 cell migration **(A)** Representative images from the wound-healing assay taken 0, 6 and 24 h after scratching. **(B)** Bar graphs plot the migration rates in DU145 and PC3 cells. * represents *P* < 0.05, ** represents *P* < 0.01.

Variation in DNA sequence can alter an oncogene's biological behavior. In order to determine if the rs61552325 polymorphism increases androgen-insensitive PCa migration, we transfected lentiviral vectors with the ERBB2 sequence containing either the C allele or G allele of rs61552325 into DU145 cells or PC3 cells, respectively. The results of western blot analyses indicate that the ERBB2 protein was overexpressed in the OE-WT (DU145 or PC3 cells infected with the major variant (C allele) virus of rs61552325) and OE-MU (DU145 or PC3 cells infected with the minor variant (G allele) virus of rs61552325) groups relative to the NC (negative control, DU145 or PC3 cells infected with the negative control virus) group (Figure 1). Using a wound healing assay, migration abilities were evaluated by measuring the distance between the two scratched edges and the migratory length of cells in the wound healing area relative to the NC group (Supplementary Table 1). Using a Student's t-test, the migratory lengths showed that 6 h after scratching, the OE-WT group (migration rate = 0.29) in PC3 cells had a significantly reduced migration ability compared to either the NC group (*P* = 0.042, migration rate = 0.48), the CON (control group, DU145 or PC3 cells without any viral infection) group (*P* = 0.004, migration rate = 0.48) or to the OE-MU group (*P* = 0.016, migration rate = 0.37) (Figure 2B). PC3 cells transfected with any of the vectors had completely healed up after 24 h, giving them a migration rate of 100% (Figure 2A). Overexpressing or mutating endogenous rs61552325 didn't significantly alter the migration activities of DU145 cells after 6 h. However, the OE-WT group (migration rate = 0.28) in DU145 cells exhibited slightly elevated migration (*P* = 0.080) when compared to the OE-MU group (migration rate = 0.13) (Figure 2B). After 24 h, both the OE-WT group (*P* = 0.005, migration rate = 0.92) and the NC group (*P* = 0.008, migration rate = 0.95) had distinctly faster migration rates relative to the CON group (migration rate = 0.59). However, there was no significant difference between the NC group and the OE-WT group (*P* = 0.914). These findings support the hypothesis that rs61552325 is a critical factor in the migration of PCa.

### ERBB2 increases androgen-insensitive PCa cell invasion and the rs61552325 minor variant decreases DU145 cells invasion

**Figure 3:**
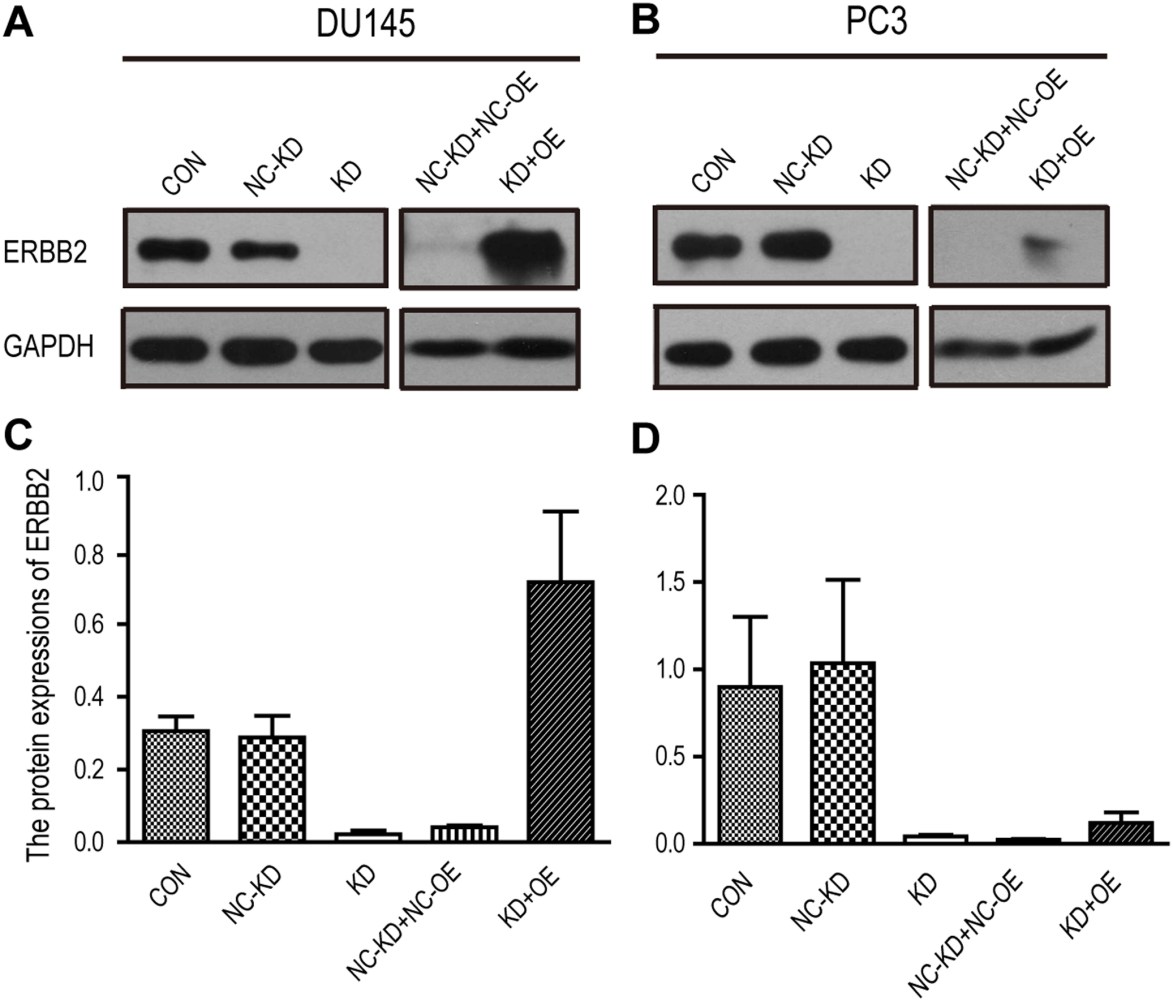
Western blot analysis of ERBB2 expression in DU145 and PC3 cells from the transwell assay **(A)** Western blot of ERBB2 in DU145 cells. **(B)** Western blot of ERBB2 in PC3 cells. **(C)** ERBB2 expression in DU145 cells. **(D)** ERBB2 expression in PC3 cells. Abbreviations: CON: control group, DU145 or PC3 cells without any viral infection; KD: DU145 or PC3 cells infected with a lentivirus containing shRNA-binded ERBB2; NC-KD: negative control knockdown, DU145 or PC3 cells infected with the negative control lentivirus against the KD group; KD+OE: DU145 or PC3 cells infected with a lentivirus containing a short hairpin RNA that binds ERBB2 and a lentivirus overexpressing the minor variant (G allele) of rs61552325; NC-KD+NC-OE: DU145 or PC3 cells infected with a negative control lentivirus against the KD group and a negative control lentivirus against the OE group.

**Figure 4:**
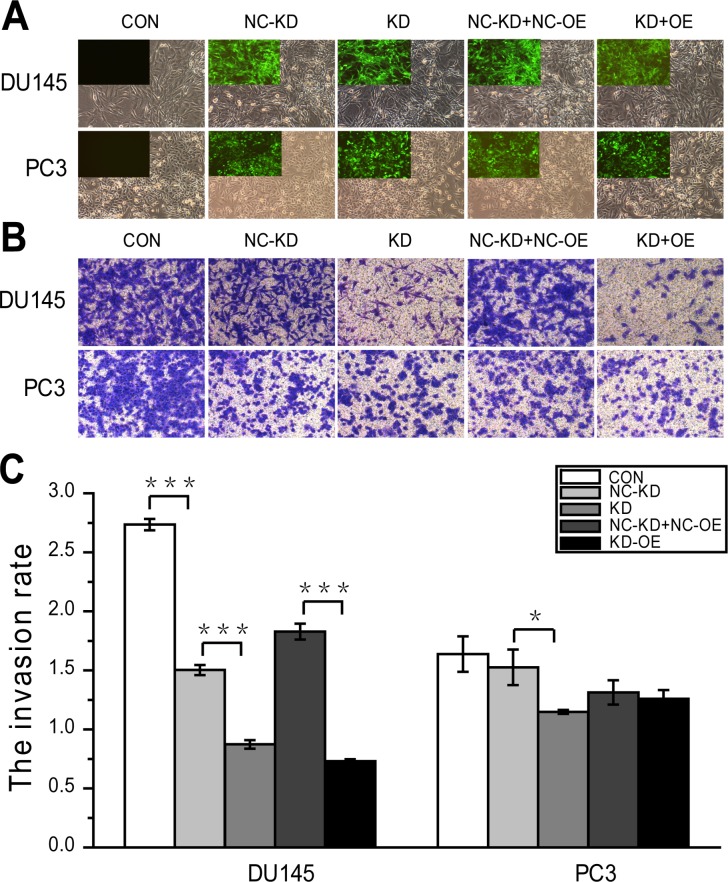
Effects of ERBB2 and the SNP rs61552325 on DU145 and PC3 cell invasion Invasive potential of different groups in DU145 and PC3 cells were measured by transwell assay after transfecting with the indicated lentiviral vectors. **(A)** Expression of GFP in DU145 and PC3 stable cell lines after infection with the indicated lentiviral vectors and selection with puromycin (immunofluorescence; magnification, × 100). For each experiment, the top left panel is a representative fluorescence image and the rest of the panel is a representative bright field image; both images are from the same field. At 20% confluency, DU145 cells in 6-well plates were infected with the indicated recombinant lentiviral vectors (MOI 20) with Enhanced Infection Solution (ENi.S; Genechem, Shanghai, China) and 5μg/mL polybrene (GeneChem), and treated with 1.00 μg/mL puromycin for 48 h at 3 d post-transduction. However, PC3 cells seeded in 24-well plates reached 15% confluency with 5μg/mL polybrene and were infected with the corresponding vectors at an MOI of 10. Cells were incubated with 1.50 μg/mL puromycin for 24 h at 3 d post-transduction to get expression and stable cell lines. **(B)** Representative micrographs of various invasion cells with membranes stained with Giemsa's solution. **(C)** DU145 and PC3 cell invasion rates. In the column diagrams, the Y-axis is the invasive cell rate calculated by determining the OD570/MTT-OD490 ratio (OD570 indicates the OD at 570 nm of the stained cells; MTT-OD490 indicates the OD at 490 nm of all other cells). Bar graphs plotting the average invasion rates of a given group were repeated three times. * represents *P* < 0.05, ** represents *P* < 0.01, *** represents *P* < 0.001.

Based on results from the wound healing test, rs61552325 is associated with migration in androgen-insensitive PCa cell lines. To confirm this result, we performed a transwell invasion assay with DU145 and PC3 cells. To determine the putative effects of rs61552325 on DU145 and PC3 cell invasion, we generated an endogenous ERBB2-knockdown in DU145 and PC3 cells using a short hairpin RNA. The absence of ERBB2 protein was verified by western blotting (Figure 3). Subsequently, ERBB2-knockdown DU145 and PC3 cells were reconstituted using a lentivirus containing the rs61552325 minor variant (G allele). Green fluorescent protein (GFP) expression was observed by fluorescence microscopy in DU145 and PC3 cells 72 h post infection. The intense fluorescent signal confirmed that all lentiviral vectors were successfully transfected into DU145 and PC3 cells (Figure 4A). The transwell insert displayed different expression levels of ERBB2 leading to a variation in the potential for chemotaxis in DU145 or PC3 cells. Staining with Giemsa's solution revealed that the CON group of cells were the best at transferring to the inside surface of the membranes (Figure 4B, Supplementary Table 2). In accordance with analysis of the fluorescent, the invasion rates of the CON group were higher than the other four groups in DU145 and PC3 cells (Figure 4C). We found that loss of ERBB2 significantly reduced the invasion ability of the KD (DU145 or PC3 cells infected with a lentivirus containing shRNA-binded ERBB2) group when compared to the NC-KD (negative control knockdown, DU145 or PC3 cells infected with the negative control lentivirus against the KD) group in PC3 cells (*P* = 0.047) and in DU145 cells (*P* = 5.2E-05). This result indicates that ERBB2 can increase the invasion capabilities of DU145 and PC3 cells (Supplementary Table 2, Figure 4C). To investigate whether ERBB2 increases the invasion of these two cell lines through rs61552325, we constructed the putative rs61552325 minor variant which showed that the KD-OE (DU145 or PC3 cells infected with a lentivirus containing a short hairpin RNA that binds ERBB2 and a lentivirus overexpressing the minor variant (G allele) of rs61552325) group had significantly lower invasion (*P* = 5.9E-04) when compared to the NC-KD+NC-OE (DU145 or PC3 cells infected with a negative control lentivirus against the KD group and a negative control lentivirus against the OE) group in DU145 cells, whereas no significant differences were seen in PC3 cells (Supplementary Table 2, Figure 4C).

## DISCUSSION

PCa is a global disease, and metastatic PCa greatly increases the difficulty of treatment. Many studies have focused on researching the connection between SNPs and metastatic PCa. A case-control study researched the relationship between SNPs in cytochrome P450 family 19 subfamily A member 1 (CYP19A1) and metastatic PCa. They found that patients with the minor variant rs4775936 had decreased chances for survival and verified their discovery in two commonly studied metastatic PCa cell lines, DU145 and PC3 [29]. rs2228013 in NKX3.1 could increase the metastasis in younger PCa patients [30].

Human ERBB2 is a member of the EGFR family of transmembrane tyrosine kinase receptors. Aberrant activation of ERBB2 by gene amplification is a common cause of pathophysiology for many tumors types, such as breast and ovarian [31–32], and has been found to play a critical role in PCa [33]. Osman I *et al*. [12] demonstrated that increased serum Her-2/neu correlates with the presence of metastatic disease, and it may indicate an increased risk of death in patients with castrate, metastatic PCa. Shi Y *et al*. [13] revealed that ERBB2 may contribute to androgen independence in PCa. Elevated ERBB2 levels in androgen-dependent PCa cells was sufficient to confer androgen-independent growth and accelerate progression to androgen-independence [14]. Tome-Garcia J *et al*. [5] have shown that overexpression of ERBB2 can increase the metastatic in the androgen-insensitive PCa cells (PC3 and DU145), as evidenced by increased cell motility and increased invasiveness, but not the androgen-sensitive PCa cells. In the present study, we found that down-regulation of ERBB2 can reduce the invasive cell behavior of PC3 and DU145 cells.

Many studies have focused on the relationship between ERBB2 SNPs and PCa [34]. Some SNPs in ERBB2 related to PCa development have been reported, such as the Val655 allele [35]. In our previous study, we found that rs61552325, located in ERBB2, is strongly correlated with PCa [20]. The polymorphism rs61552325 changes an amino acid in ERBB2 from proline to alanine which might increases normal cellular signal transduction and leads to invasion of PCa. In this study, we used four types of bioinformatics software to predict the correlation of the rs61552325 mutation on ERBB2 protein stability. The results showed that the mutation Pro1140Ala would decrease the stability of ERBB2 protein and its interaction. Consequently, we verified the functional disruption caused by rs61552325 in an androgen-insensitive PCa cell line with a wound healing assay and a transwell assay. We came to the conclusion that the rs61552325 minor variant is associated with an increased risk for PCa invasion. There have been several reports in the literature about rs61552325. Xin DJ *et al*. [36] demonstrated that rs1058808 polymorphisms may be related to osteosarcoma susceptibility in the Chinese Han population. Su [37] indicated that rs1058808 polymorphisms are associated with HER2 expression in breast cancer. Song GG *et al*. [23] showed that rs61552325 is related to asthma. Tong SY *et al*. [22] found that the rs1058808 CG/GG genotype lead to a significantly increased risk of endometrial cancer in Korean women with a BMI ≥ 25 kg/m^2^ when compared to subjects with a normal BMI (*P* for linear trend < 0.05). In our previous study, we found an association between rs61552325 and PCa. Our cell function experiments showed that polymorphism significantly increased the migration of PC3 and DU145 cells. Protein structure modeling has revealed that many non-synonymous single-nucleotide variants (SNVs) have a deleterious effect on protein stability, structure and function [38]. Our bioinformatics analyses also reveal that the mutant Pro1140Ala would likely decease the stability of the ERBB2 protein and its interaction. Additionally, the rs61552325 variant slows the migration of PC3 and DU145 cells. Our study may initiate a new direction of research into invasion PCa and play a guiding role in the development of new treatments for PCa.

## MATERIALS AND METHODS

### *In silico* predictive mutational analysis

Four types of software were applied to predict the correlation of the rs61552325 mutation on ERBB2 stability and interaction. Many human genetic diseases associated with single point mutations can be predicted by I-Mutant Suite [24, 25] with Support Vector Machines and evolutionary information. The free energy change predicted with this tool is based on the Gibbs free energy change between the major variant and minor variant proteins, which can be categorized into three classes: destabilizing mutations (DDG < -1.0 kcal/mol), stabilizing mutations (DDG > 1.0 kcal/mol) and neutral mutations (-1.0 ≤ DDG ≤ 1.0 kcal/mol). iStable [26] software is an integrated predictor with grid computing architecture using sequence information. PolyPhen-2 [27] uses eight sequence-based and three structure-based predictive features selected automatically by an iterative greedy algorithm for predicting damaging missense mutations. HOPE [28] collects information from a wide range of information sources including calculations on the 3D coordinates of a protein from the UniProt database and predictions by Distributed Annotation (DAS) services, and analyzes the structural and functional effects of point mutations.

### Cell culture and treatments

DU145 cells were purchased from Wuhan Boster Bio-Engineering Limited Company (Wuhan, China) and PC3 cells were purchased from Xiangya Central Experiment Laboratory, Central South University (Changsha, China). The DU145 and PC3 cell lines were cultured *in vitro* in RPMI 1640 (Invitrogen, Melbourne, Australia) supplemented with 10% fetal bovine serum (FBS; GIBCO). The media was refreshed every day in order to maintain cell growth. All cell lines were incubated at 37°C with 5% CO_2_ in a sterile incubator (SANYO, MCO-175, Japan).

### Construction of lentiviral vectors

Major variant and minor variant vectors of rs61552325 were constructed to explore the association of this SNP with cell migration in PCa. PCR fragments containing either the C or G allele of rs61552325 were amplified from genomic DNA isolated from homozygous C or homozygous G carriers of SNP rs61552325 using the following primers: forward primer (5′-AGGTCGACTCTAGAGGATCCCGCC ACCATGGAGCTGGCGGCCTTGTGCCGCT-3′), reverse primer (5′-AGTCCATGGTGGCGACCGGCACTGGCACGTCCAGACCCAGGTACTCTG-3′). The two types of lentiviruses constructed were generated by inserting the corresponding PCR product into the BamHI/AgeI digested retroviral vector GV166-MCS-Ubi-EGFP (GeneChem). Further validation that the cloned PCR fragments contained the expected C or G allele of rs61552325 was conducted through DNA sequencing technology. For binding the ERBB2 sequence (5′-TCAGTATCCAGGCTTTGTA-3′), we cloned the corresponding oligonucleotides for a short hairpin RNA -encoding sequence into a HpaI/XhoI digested retroviral vector GV118-U6-MCS-Ubi-EGFP (GeneChem). In the presence of lipofectamine 2000 (Invitrogen, Carlsbad, CA, USA), lentiviral packaging plasmids (GeneChem) and our designed vectors were separately co-transfected into 293T cells, which were seeded onto 20 cm plates in the previous day. Lentivirus-containing supernatants were harvested 48 h after transfection.

### Lentiviral transfection

DU145 and PC3 cells were incubated in 6-well plates at a density of 1×10^5^ cells/well containing 2 mL of serum-free growth media prior. At 15% confluence, cells were transfected with lentivirus at the optimal multiplicity of infection (MOI) of 10 or 20, and cultured at 37°C in 5% CO_2_ for 12–16 h. Subsequently, the supernatant was discarded and growth media containing serum was added. For transwell invasion, 72 h after transduction into PC3 and DU145 cells, lentiviruses were selected with puromycin at 1.50 μg/mL for 24 h and at 1.00 μg/mL for 48 h, respectively. Lentiviruses for the scratch test were selected 48 h after transduction with puromycin at 3.00 μg/mL for 48 h. Surviving and stable transfectants were expanded to make clonal cell populations for further study. In order to ensure transduction efficiency for the transwell invasion assay, the expression levels of GFP from lentiviral vectors were detected by inverted fluorescence microscopy (micropublisher 3.3RTV Olinpass Company) 4 d post-transduction.

### Wound healing assay

A wound healing assay allows researchers to study cell migration and cell interactions. DU145 or PC3 cells were seeded into each well of a 96-well plate at 3×10^4^ cells/well and infected with the designated lentiviral vectors. The following day wound gaps were made by scraping cell monolayers with a scratch tester in the center of the well, the cells were washed with serum-free medium twice, and then media containing 2% FBS was added. The cells were cultured for 24 h at 37°C with 5% CO_2_. To evaluate wound closure, images of the wound edges were acquired at the same location 0, 6 and 24 h after wounding. Each observation was carried out in triplicate at three different locations per well.

### Transwell invasion assay

The transwell invasion assay was based on a previous report [39–41]. Briefly, DU145 and PC3 cells transfected with lentiviral vectors were plated 5 days post-transfection in 100 μL of serum-free media with 1×10^5^ cells per transwell (3422 Corning, China) and allowed to transfer to the lower chamber which contained 600 μL of RPMI 1640 media supplemented with 30% FBS (Invitrogen). After incubation for 24 h, non-invasive cells on the upper surface were completely removed with a cotton swab. Cells that had migrated to the lower surface of the insert were stained with Giemsa's solution for 20 min. Images of the membranes were captured under a microscope (micropublisher 3.3RTV Olinpass Company). Then, to test the optical density at 570 nm (OD570), the membranes were dissolved in 10% acetic acid. Each cell line was plated in triplicate and each experiment was repeated three times.

### Western blotting of ERBB2

Total protein was extracted using the Radio-Immunoprecipitation Assay (RIPA) lysis buffer. Protein samples were resolved using 10% sodium dodecyl sulfate polyacrylamide gel electrophoresis (SDS-PAGE) followed by electrophoretic transfer to a polyvinylidene fluoride membrane. The membranes were probed with an IgG anti-Rabbit ERBB2 monoclonal antibody (ABCAM, China), followed by incubation for 2 h at room temperature in a 1:5000 dilution of secondary antibodies against rabbit IgG conjugated to horseradish peroxidase (Santa Cruz). Protein bands were detected using the electrochemiluminescent (ECL) detection system (Zhongshan Golden Bridge Bio Technology, China) and GADPH staining served as a loading control. Protein expression levels were calculated with Image J software (National Institutes of Health).

### Statistical analysis

All experiments in the study were repeated three times. Means were compared using a Student's t-test. *P* value of less than 0.05 were considered to be statistically significant. All of the data was analyzed by SPSS statistical software (version 16.0).

### Limitation

Although we conducted a wound healing assay and transwell invasion assay, we did not conduct an apoptosis experiment. All of our experiments were conducted in two typical metastatic PCa cell lines and perhaps other cell lines might behave differently. We have only identified one locus and have not taken the chain site influence into consideration. Thus, further research needs to be done to confirm this association.

## SUPPLEMENTARY MATERIALS TABLES






