# Supplementary material for: Functional analysis implicating the SNP rs61552325 in ERBB2 as an effector for androgen-insensitive prostate cancer cell invasion
Source: Oncotarget. 2017 Apr 4;8(20):33745–55. doi: 10.18632/oncotarget.16807 (PMC5464908; doi:10.18632/oncotarget.16807)
Supplement: Supplementary file 1 [file oncotarget-08-33745-s001.pdf]

Functional analysis implicating the SNP rs61552325 in ERBB2 as an effector for androgen-insensitive prostate cancer cell invasion

SUPPLEMENTARY TABLES

Supplementary Table 1: Migrate rates of PC3 and DU145 cell lines by wound healing assay

See Supplementary File 1

Supplementary Table 2: Invasion rates of PC3 and DU145 cell lines by transwell assay

See Supplementary File 2
